# The Characteristics of Steamed Bread from Reconstituted Whole Wheat Flour (WWF) of Different Hard Wheat Classes with Different Bran Particle Size Distributions

**DOI:** 10.3390/foods10102413

**Published:** 2021-10-12

**Authors:** Yuching Huang, Andrew L. Mense, Lingzhu Deng, Meiying Su, Kuenho Shih, Jayne E. Bock

**Affiliations:** 1 China Grain Products Research & Development Institute (CGPRDI), New Tapei City 24941, Taiwan; ching.huang@CGPRDI.org.tw (Y.H.); kh.shin@CGPRDI.org.tw (K.S.); 2 Wheat Marketing Center, Portland, OR 97209, USA; amense@wmcinc.org; 3 Department of Food Science, Technology at the Ohio State University, Columbus, OH 43210, USA; deng.869@osu.edu; 4 Agricultural Technology Research Institute (ATRI), Hsinchu City 300110, Taiwan; 1101001@mail.atri.org.tw

**Keywords:** reconstituted whole wheat flour, bran, bran particle size distribution, steamed bread

## Abstract

The purpose of this study was to investigate the effects of reconstituted whole wheat flour (WWF) particle size on flour characteristics and northern-type steamed bread (NTSB) quality. In this study, hard white (HW), hard red winter (HRW), and hard red spring (HRS) wheat classes, and four different bran particle size distributions [D(50) values of 53 μm, 74 μm, 105 μm, and 125 μm] were blended at a ratio of 85% refined flour + 15% bran to create reconstituted WWF and make reconstituted WWF NTSB. Farinograph water absorption and water solvent retention capacity (SRC) increased as bran particle size decreased. Flour and dough strength tests such as lactic acid SRC and Farinograph and Mixolab development time and stability did not show any clear trends with bran particle size. HRW WWF tended to be the exception as Farinograph development time and stability generally increased as particle size increased. Resistance to extension increased as bran particle size decreased for HRW WWF and increased as particle size increased for HW and HRS. These differences in WWF dough rheology trends were likely due to differences in gluten characteristics between the classes. The results showed that larger particle sizes (105 μm and 125 μm) were more conducive to achieving desirable whole wheat NTSB specific volume, color, and texture.

## 1. Introduction

Chinese steamed bread (CSB) is a popular food in China and many Asian countries [1]. CSB is a fermented wheat flour product that is produced in a similar manner to many Western breads [2,3]. The steaming step imparts a white and smooth surface in contrast to a crust formed during baking at high temperatures [2,3]. It originated in and is a staple of northern China [4,5]. The two major types of Chinese streamed bread are the northern-type steamed bread (NTSB) and southern-type steamed bread (STSB) [6]. The texture and eating quality of the northern style is described as cohesive, dense, firm, chewy, and elastic, with an even distribution of small cells in the crumb [2,3,5]. The southern-style texture is known for its open and soft texture with a very white, shiny, and smooth appearance [2,3,6]. Another difference is that northern-style steamed bread has less sugar than the southern-style [4]. Because of its simple formula, changing the type of flour may change the quality of northern-style steamed bread.

Steamed bread accounts for approximately 40% of China’s wheat usage, and its market has spread to North America and Europe [2,7]. Taiwan’s Department of Statistics points out that the annual output value of baked and steamed food is about 6.2 billion NT dollars [8]. Therefore, the incorporation of whole wheat flour (WWF) or bran would increase dietary fiber levels and provide health benefits to a wide population [9].

WWF refers to flour that uses 100% of the wheat kernel, including all bran, germ, and endosperm materials [10]. Whole wheat is a good source of dietary fiber, minerals, vitamins, and antioxidants [11,12]. The consumption of whole grain products is associated with a decreased risk developing of obesity, type 2 diabetes, cardiovascular disease, and cancer [11]. However, the finished product quality and sensory challenges associated with the incorporation of WWF into a baked good influence public acceptance [5,11].

The presence of the bran and germ results in darker product color, reduced product volume, and firmer texture for products containing WWF [9]. These effects have been studied in a wide variety of finished products such as bread, noodles, crackers, pasta, flatbread, and tortillas [2,13]. Information on how WWF and bran characteristics such as particle size affect dough properties and finished quality of CSB is limited and needs further investigation [5,7,12,13].

Using WWF to replace refined flour is not always simple. Whole wheat flour comprised of coarse bran produced CSB with better texture and crumb structure and equivalent specific volume, skin color, smoothness, and shape compared to a fine bran WWF [14]. Xu et al. [12] reported that decreasing WWF particle size decreased specific volume and resulted in firmer crumbs for both STSB and NTSB. In contrast, Wang et al. [2] found that coarse WWF produced Chinese STSB with smaller specific volume, firmer texture, less open crumb grain, thicker cell walls, and a more compact structure compared to a finer WWF.

The effects of bran particle size on water absorption and dough rheology are also varied. It has been reported that water absorption for fine bran WWF was greater [12,14] and lesser [2,13] compared to coarse bran WWF. Zhang and Moore [15] found that bran particle size did not affect Farinograph dough water absorption, although the same authors reported coarse bran to have higher water retention capacity than finely ground bran [15]. Reduced bran particle size was found to both decrease dough mixing time [2,12,13,15] and increase it [16] compared to coarse bran WWF. In addition, fine bran WWF has been reported to result in both a decrease in dough stability [15] and an increase in stability [2,13,14,16] compared to coarse bran WWF. Mixolab starch hot gel stability and retrogradation were found to increase with a reduction in WWF particle size [2,12,13]. A reduction in WWF particle size was also found to increase dough strength [2,15].

These effects on volume, rheology, and texture may be explained by studies that have indicated that the presence of bran in dough not only affects gluten structure [9,17], but also affects gas cells in the dough [9,18,19]. Therefore, the bran fraction is considered to be an important factor affecting the structure and properties of bread [19,20,21].

Another factor affecting the baking quality of WWF bread is the variability inherent in wheat bran. The addition of bran is challenging because it does not have a consistent chemical composition and quality [15]. Wheat bran composition can vary due to wheat class, variety, growing environment, grain shape and size, the thickness of bran layers, and the milling technique used [7,11,15]. Roller milling systems can be used to produce recombined WWF [22]. The endosperm is separated from the bran and germ allowing for the endosperm to be gradually reduced and later recombined with bran or with components that represent the entire wheat kernel [10,22]. Roller mills are more flexible than other milling systems such as hammer mills and stone mills due to the ability to separate out the bran and germ for additional treatment such as particle size reduction, hydration, fermentation, and heat treatment [5,7,10,22]. Roller mills produce less heat than a stone mill during the whole wheat flour milling process due to the use of cooling systems for the rollers, which results in less altering of the starch, protein, and lipid fractions [5,22].

In this study, three different classes of wheat [hard white (HW), hard red winter (HRW) and hard red spring (HRS)] and four different particle size distributions of bran [D(50) values of 53 μm, 74 μm, 105 μm, and 125 μm] were blended at a fixed ratio (85% refined flour + 15% bran) to create reconstituted WWF and make reconstituted WWF NTSB. The purpose of this study was to investigate the effects of reconstituted WWF particle size on flour characteristics, dough rheology, and NTSB quality. The results show that larger particle sizes produce NTSB with more desirable quality attributes than smaller particle sizes.

## 2. Materials and Methods

### 2.1. Materials

Wheat from three U.S. wheat classes (HW, HRW, and HRS) was used in this study. Their protein contents (dry basis) were 15.1% (HW), 13.8% (HRW) and 17.1% (HRS). The three wheat samples were tempered in plastic buckets at room temperature to a final moisture level of 14.5% and milled on a Miag Multomat experimental mill (Bühler Inc., Plymouth, MN, USA). The refined flours (HWf, HRWf, and HRSf) were the controls in this study. Bran from each respective wheat sample was milled by Prater Industries (Bolingbrook, IL, USA) on a laboratory scale air classifier mill to four particle size distribution targets with D(50) values of 53 μm, 74 μm, 105 μm, and 125 μm. Other materials included instant dry yeast (Lesaffre Yeast Corp., Milwaukee, WI, USA), sugar, salt, and shortening (Crisco, J.M. Smucker Co., Orrville, OH, USA).

### 2.2. Preparation of Reconstituted Whole Wheat Flours (WWF)

After milling, control flours (HWf, HRWf, and HRSf) were mixed with bran from each particle size distribution target at a ratio of 85% refined control flour to 15% bran (*w*/*w*) to create reconstituted whole wheat flour (WWF). The final sample set included: HW (HWb53, HWb74, HWb105, and HWb125), HRW (HRWb53, HRWb74, HRWb105, and HRWb125), and HRS (HRSb53, HRSb74, HRSb105, and HRSb125). This study utilized reconstituted whole wheat flours to control variability stemming from attempting to combine different ratios of bran and shorts from the wheat classes studied.

### 2.3. Flour Composition and Quality Analyses

All samples were analyzed for moisture, protein, ash and damaged starch contents according to AACC Approved Methods (44-15A; 08-01; 46-30; 76-33.01, respectively) [23]. Because the SD-matic (Chopin Technologies, Villeneuve-la-Garenne, France), an amperometric method, has not been evaluated for measurement of starch damage in WWF, an enzyme-based starch damage assay kit (Megazyme, Chicago, IL, USA) was also used to quantify starch damage using AACC Method 76-31.01 [23]. The amperometric method (AACC 76-33) uses the absorption kinetics of iodine, as measured by an amperometric probe, to express the damaged starch content. The other method (AACC 76-31) is based on enzymatic hydrolysis under given temperature, pH, and enzyme conditions. In that method, α-amylase acts on the flour sample to break down the starch into maltosaccharides and dextrins, and amyloglucosidase breaks these sugars down to glucose. Starch damage is calculated according to the amount of reducing sugars produced.

Wet gluten content (AACC 38-12.01) is commonly used to indicate quality differences, especially among flours with similar protein contents [24], and is particularly valuable in discriminating durum semolina quality [25,26]. Control flours presented with wet gluten values (14% mb) on the order of HRS (34.7 ± 0.0) > HW (32.8 ± 0.1) > HRW (26.5 ± 0.3), which closely tracks with their respective protein contents. However, screen flooding was encountered for the WWFs with smaller bran particle size distributions and thus WWF wet gluten contents were not collected. SRC profiles were recorded as an alternative to wet gluten because SRC profiles, and especially the gluten performance index (GPI) calculated from those profiles, have been more definitively linked to end product quality of refined and WWFs by various authors [27,28,29,30,31].

To evaluate the flour quality characteristics (polymer swelling, water absorption, stability, starch gelatinization, retrogradation, and extensibility), Solvent Retention Capacity (SRC) (AACC Method 56-11.02), Farinograph (AACC Method 54-21.01) (C.W. Brabender, South Hackensack, NJ, USA), Mixolab (AACC Method 54-60.01) (Chopin Technologies, Villeneuve-la-Garenne, France) and Kieffer rig extensibility (Texture Technologies, Hamilton, MA, USA) tests were conducted [23]. The Kieffer rig extensibility test was partially modified according to the method described by Londono et al. [32]. Dough samples were prepared as appropriate for full formula steamed bread production. After dough sheeting, the dough was flattened with a rolling pin and placed on a grooved base with a Teflon coating. A flat-top piece was applied to the dough on the grooved base and pushed down firmly by tightening a clamp until the two pieces came together to separate the dough into strips. The mold and clamp apparatus was then placed in a closed plastic bag to allow the dough to relax for 45 min (30 °C, 85% RH). After the relaxation period, the clamp and flat top piece were removed and the dough strips were removed for extensibility measurement on the TA.XTPlus (Texture Technologies, Hamilton, MA, USA). The test parameters were pretest speed, 2.0 mm/s; test speed, 3.3 mm/s; post-test speed, 10 mm/s; trigger force, 5 g; and data acquisition rate, 200 PPS.

### 2.4. Steamed Bread Preparation

**Figure 1:**
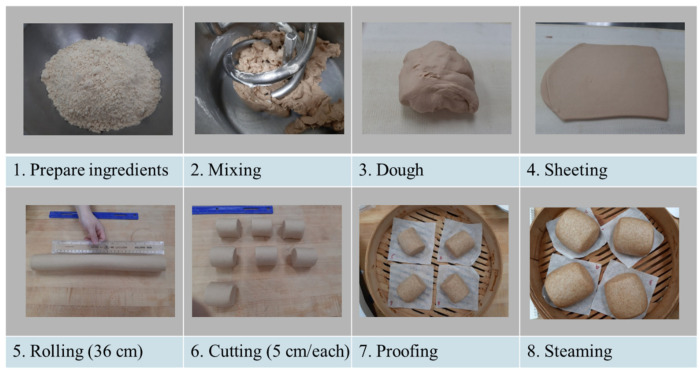
Steamed bread making procedure.

The steamed-bread making process is outlined in Figure 1. The formula included: 400 g flour, 8 g instant dry yeast, 180–200 g water [optimized based on Farinograph water absorption (WA)], 32 g sugar, 1.2 g salt, and 16 g shortening. The yeast, sugar, and salt were each dissolved separately in water. The mixtures were then added to the flour and mixed in a Hobart mixer (Model A-120, Hobart Manufacturing Co., Troy, OH, USA) equipped with a special double spiral head at speed 1 for 1 min. The shortening was then added and mixed into the dough at speed 1 for another 1.5 min. The final dough was rested in a plastic bag for 10 min before sheeting until the surface was smooth (10–12 passes) using an Oshikiri sheeter/molder (Model WFS, Oshikiri Machinery Ltd., Kanagawa, Japan). The dough sheet was rolled into a cylinder by hand to 36 cm and cut into 6 equal parts (5 cm/piece). One piece of dough (25 g) was placed in a 45 mL plastic centrifuge tube before proofing. Initial dough volume was 21–22 mL. The dough pieces were then proofed (30 °C, 85% RH) in a proofing cabinet (Model LRPR-2, LBC Bakery Equipment, Everett, WA, USA) until the volume of dough in the centrifuge tube reached 40–45 mL. The remaining 5 pieces of dough were steamed for 15 min using a convection oven (Model SCCWE 62G, Rational AG, Landsberg am Lech, Germany).

### 2.5. Quality Evaluation of Steamed Bread

All steamed bread samples were evaluated for specific volume, exterior color, and texture. The specific volume of steamed bread was determined using a laser volume analyzer (BVM-L370, TexVol Instruments Inc., Viken, Sweden) and dividing the measured volume by the weight of the bread piece. The exterior *L*, *a*, *b* color was determined using a chroma meter (CR-410, Konica Minolta Sensing Inc., Tokyo, Japan). The texture profile analysis (TPA) of the steamed bread was determined using the TA.XTPlus Texture Analyzer equipped with a 35 mm acrylic cylindrical probe. Steamed bread was sliced horizontally, and a flat piece of 15 mm thickness was compressed to 50% of its original height in a two-compression cycle. The test conditions were pretest speed, 2 mm/s; test speed, 1 mm/s; post-test speed, 1 mm/s; and trigger force, 5 g.

### 2.6. Statistical Analysis

All measurements were performed at least in duplicate. A one-way Analysis of Variance (ANOVA) was performed using SAS statistical analysis software. Duncan’s test was used for the comparison of means. The value *p* < 0.05 was used to determine significant differences between mean values. The results of the study were presented as the average ± standard deviation.

## 3. Results and Discussion

### 3.1. Compositional Analysis

Table 1 shows the moisture, protein, ash, and damaged starch contents of the bran, refined flours, and reconstituted WWF used in this study. The moisture content of the control flours (HWf, HRWf, and HRSf) was 13.00–13.75% and consistently greater than that of the reconstituted WWF (12.25–12.74%). This is reasonable as the bran had a moisture content of 6.69% and would naturally reduce the average moisture content of the reconstituted WWF. The lower moisture content of reconstituted WWF compared to a refined flour control was also reported by Liu et al. [5]. Ash content was greater for the reconstituted WWF, as expected, due to the presence of bran (Table 1), which aligns with previous research [5]. However, there were few significant differences in protein content on the addition of bran. The HRW reconstituted WWF had significantly greater protein content than the refined flour (Table 1).

Damaged starch content of the control flours varied depending on wheat class, likely due to differences in kernel texture and milling characteristics [10]. Starch damage, in order from greatest to least, was as follows: HRWf (5.92%) > HRSf (4.35%) > HWf (3.23%) (Table 1). Surprisingly, adding 15% bran only had a small effect on the damaged starch content within the same class of wheat. Regrinding of bran normally increases starch damage in the residual endosperm removed with the bran fraction. It is possible that the air classification strategy used to regrind the bran for this study may have improved bran passage through the screen, thereby minimizing excessive grinding and subsequent starch damage.

### 3.2. Damaged Starch Content

Damaged starch is one of the most important factors affecting quality characteristics of flour [33]. There are two approved methods for determining damaged starch as distinguished by their measurement principle: amperometric (SD-matic) and enzymatic hydrolysis (Megazyme) methods.

A comparison of the starch damage results obtained from each method are presented in Table 2. Looking at the refined flours, all samples exhibited lower starch damage using the SD-matic compared to the Megazyme method. It is apparent that the results were influenced by the measurement method, and this held for refined flour as well as WWF. Interestingly, the results from both methods appeared to be correlated. The general ranking of samples did not change between the two methods. That is, the order of starch damage from greatest to least within a wheat class was generally similar for both methods. Another finding was that the addition of bran, regardless of particle size, resulted in a generally consistent level of starch damage when an air classified grinding process was used to reduce bran particle size (Table 2). In contrast, the starch damage of WWF was reported to increase as bran particle size was reduced [34,35]. The WWF starch damage (Table 2) was incrementally lower for HRW and HRS and greater for HW compared to their refined flour control. Niu et al. [35] reported that superfine grinding of bran increased WWF starch damage compared to straight grade flour while Wang et al. [34] found that WWF starch damage was not greater than straight grade flour.

Whereas the Megazyme method is considered a direct method for damaged starch measurement, the amperometric method utilized by the SD-matic is indirect. Thus, a slight algorithm correction to the SD-matic may be necessary to pull these results into better alignment. Another factor to consider is that the SD-matic mechanism is based to some degree on the formation of an iodine-amylose complex. Therefore, the amylose content of the flours could be a factor that contributed to the shift towards lower starch damage values for the SD-matic [36]. McAllister et al. [36] reported that compared to the mean amylose content of the flours used in their study, flour samples with higher amylose were generally overpredicted and flour samples below this mean value were underpredicted. Overall, given the advantages of the SD-matic (shorter test time, fewer reagents, less technical skill required), it is an acceptable means of differentiating the damaged starch content of WWF in industrially relevant settings.

One final observation is that the refined flours for HRW and HRS exhibited slightly greater starch damage than their WWF counterparts across all bran particle size distributions. This finding was unexpected and does not match the results for HW refined and WWFs. If this were an issue of dilution due to the presence of bran, we would expect to see it manifest across all three wheat classes. As the bran fraction used for reconstitution contained only the outermost bran layers, our observations may be more a function of bran differences among wheat classes and how they affect fractionation into different mill streams. Indeed, Hemery et al. [37] reported differences in bran mechanical properties and composition between soft and hard wheat that could potentially affect grinding behavior.

### 3.3. SRC Profiles

The characteristic dense and firm texture of NTSB is achieved primarily by using a 10–12% protein hard wheat flour exhibiting medium to high dough strength [3,12]. SRC is a diagnostic test that can help delineate to what extent gluten, damaged starch, and arabinoxylans (AX) contribute to flour functionality and end product quality [38]. SRC values, including water SRC (W-SRC), lactic acid SRC (LA-SRC), sodium carbonate SRC (SC-SRC), and sucrose SRC (Su-SRC), are shown in Table 3.

Water SRC (W-SRC) is influenced by the swelling of gluten, damaged starch, and AX [38], and it provides an overall picture of water requirements for a given flour. The W-SRC values increased with the addition of bran in agreement with previous reports [34,39] (Table 3). The W-SRC values were greater for WWF with fine particle size bran (Table 3). It is likely that the larger surface area to volume ratio of smaller bran particles allowed for increased water uptake. In contrast, Wang et al. [34] found that grinding bran and shorts to a finer particle size did not affect WWF W-SRC.

Lactic acid SRC (LA-SRC) is specific for swelling of gluten polymers, specifically glutenins, with greater values potentially indicating stronger gluten and greater protein functionality. The protein contents of HWf, HRWf, and HRSf were 13.77%, 12.36%, and 15.66%, respectively (Table 3). The LA-SRC values were on the order of HRSf > HRWf > HWf, indicating that although HWf had a higher protein content than HRWf, the gluten strength was lower than HRWf. The WWF LA-SRC values from largest to smallest were aligned with the refined flour trends HRS > HRW > HW (Table 3). After adding bran, the LA-SRC values of all classes decreased compared with their control groups (Table 3) in line with Wang et al. [34] and Bettge et al. [39]. The decrease could be ascribed to the dilution of functional gluten proteins by bran due to less gluten being present by weight compared to the refined flour [34,39]. Bran particle size did not greatly impact overall LA-SRC in agreement with Wang et al. [34] or GPI values (Table 3). It, therefore, appears that the effect of bran on LA-SRC is more dependent on its presence rather than its particle size. Interestingly, the GPI was greater for HRW than HW despite the greater protein and wet gluten contents of the HW flours. Indeed, Maghirang et al. [40] reported that wet gluten content and gluten index were not capable of predicting quality differences between U.S. HRW and HRS wheats. It appears that GPI may be a better indicator of gluten quality than tests like wet gluten content and gluten index, as other authors have also suggested [38].

Sucrose SRC (Su-SRC) evaluates the functional contribution of AX to flour and baking performance. The bran contains more AX than the endosperm [41], and hence the reconstituted WWF had greater values than the control flours (Table 3), consistent with Bettge et al. [39]. However, the exception was the HRW WWF, where nearly all samples had similar Su-SRC values to the control. Different bran particle sizes did affect the value of Su-SRC, with smaller bran particles showing increased Su-SRC values. The AX in smaller bran particles, by virtue of the greater surface area to volume ratio, may be more accessible to the solvent than those in larger bran particles.

Sodium carbonate SRC (SC-SRC) provides information on the functional contribution of damaged starch to the flours. The SC-SRC values of the control groups (HWf, HRWf, and HRSf) showed that the contribution of damaged starch from low to high was HWf < HRSf < HRWf (Table 3). This aligned with the starch damage values reported in Table 1 and Table 2. The reconstituted WWF SC-SRC values for HW, HRW, and HRS were greater than the control groups, in agreement with Wang et al. [34] and Bettge et al. [39]. The higher values do not necessarily indicate that the WWF samples had greater levels of starch damage as SC-SRC is merely a means to determine the contribution of starch damage to overall polymer swelling, and thus its contribution to flour functionality and baking performance [34]. The accessibility of the SC-SRC solvent to flour components that swell in its presence also affect the results, and this accessibility is perhaps more important than total starch damage per se. Bran particle size did not affect SC-SRC (Table 3). Within the WWFs, the SC-SRC values from low to high were HW < HRS and HRW, which generally agreed with Table 1 and Table 2. The Megazyme and SD-matic starch damage results for the WWFs from low to high were HW < HRS < HRW (Table 1 and Table 2).

### 3.4. Farinograph Characteristics

Table 4 shows the Farinograph results for the refined flours and reconstituted WWF. The water absorption (WA) of HW, HRW, and HRS increased when 15% bran was added compared with controls (Table 4), which aligns with W-SRC results (Table 3). The increase in Farinograph WA with the addition of wheat bran has been widely reported [5,16,35,42]. It is a result of the high fiber content of wheat bran and the presence of hydroxyl groups that interact with water through hydrogen bonding [16,35]. Farinograph WWF WA from high to low was HRS > HRW > HW. The WWF W-SRC results had HRW as having a slightly higher WA than HRS, whereas HW had the lowest absorption (Table 3). Farinograph water absorption increased as bran particle size decreased for the HW, HRW, and HRS reconstituted WWF samples (Table 4), which likely corresponds to the greater surface area to volume ratio. These results also align with the W-SRC results (Table 3). It should be noted, however, that the effects of bran particle size on Farinograph WA have been varied. Farinograph water absorption has been reported to be independent of bran particle size [15,43], increase with an increase in particle size [16,42], and increase with a decrease in particle size [14,35].

The refined flour development time (DT) and stability (ST) were on the order of HRSf > HWf > HRWf (Table 4). This order did not directly translate to the WWF Farinograph results. The WWF Farinograph ST from longest to shortest was HRW > HRS > HW (Table 4). Interestingly, HRS WWF had the greatest LA-SRC (Table 3), indicating greater gluten strength but not the longest ST (Table 4). HW WWF had the lowest LA-SRC, which aligned with the shortest DT and ST. The HW and HRS WWF DT and ST were shorter compared to the refined flours. The HRW WWF group proved to be the exception to this trend. After adding 15% bran, the DT and ST increased compared to the refined control (Table 4).

The effect of bran on Farinograph DT and ST compared to a refined flour has been varied. Farinograph DT has been reported to be reduced for coarse WWF compared to refined flour [16,35,42] while increased for fine WWF [16,42]. However, Niu et al. [35] found that fine WWF reduced DT compared to a refined flour. Farinograph ST was found to decrease for coarse, medium, and fine WWF particle sizes compared to a straight grade flour control [35,42]. In contrast, WWF with a fine particle size resulted in an increase to ST compared to a refined flour [16].

The HRW WWF DT and ST tended to increase as bran particle size increased. It may be that HRW bran provided structural reinforcement, thereby increasing dough resistance against the mixing blades and delaying curve departure from 500 BU as observed by other researchers [44,45]. The HW and HRS WWF DT and ST did not show any strong trends with bran particle size (Table 4). The effect of WWF particle size on Farinograph DT and ST has been varied. A reduction in WWF particle size has been reported to increase DT and ST [16,42], decrease it [15], and have little effect on DT and ST [35].

Milling technique and mill flow, whole wheat and bran particle size reduction strategy (e.g., directly grinding wheat into WWF, reconstituting WWF, reduction of bran particle size before reconstituting with refined flour, etc.), wheat genetics, and growing conditions are several factors that influence Farinograph WA, DT, and ST results. The lack of agreement in the literature is likely a combination of these factors. Establishing a standardized method for whole wheat flour milling would resolve the extrinsic factors contributing to conflicting flour quality results, leaving the remaining variability to intrinsic factors such as genetics and environment.

### 3.5. Mixolab Characteristics

**Figure 2:**
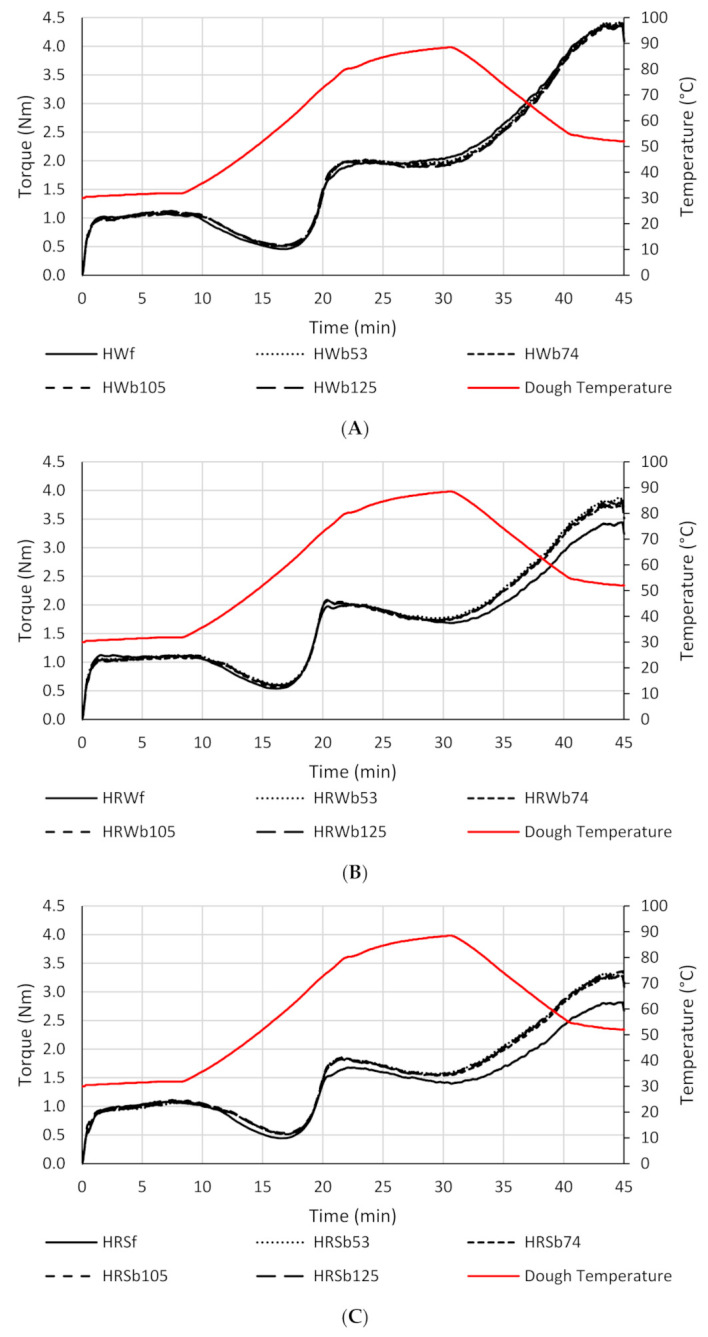
Mixolab curves of refined flours and reconstituted whole wheat flour (WWF) of hard white (HW), hard red winter (HRW), and hard red spring (HRS) wheat classes. HWf, HRWf, and HRSf were 100% refined straight grade control flours. Reconstituted WWF contained 85% refined control flour and 15% bran (*w*/*w*). Reconstituted WWF: HWb53, HRWb53, and HRSb53 with an average bran particle size of 53 μm; HWb74, HRWb74, and HRSb74 with an average bran particle size of 74 μm; HWb105, HRWb105, and HRSb105 with an average bran particle size of 105 μm; HWb125, HRWb125, and HRSb125 with an average bran particle size of 125 μm. (**A**), HW; (**B**), HRW; (**C**), HRS.

Mixolab results for the refined and reconstituted WWF are shown in Table 5 and Figure 2A–C. C1 measures the time required for flour to reach peak development while ST represents how long the dough is stable during mixing at 30 °C with a longer time indicating a stronger flour. These parameters are somewhat analogous to DT and ST in the Farinograph, although there are significant differences between the tests (i.e., mixing geometry, mixing speed, total dough mass, etc.) [46]. The WWF Mixolab ST from longest to shortest was HRW > HW > HRS (Table 5). The Mixolab ST values did not exactly match the Farinograph results (Table 4). Unexpectedly, the HRS WWF had the shortest ST (Table 5). Similar to the results from the Farinograph, the addition of bran to HRWf resulted in an increase in C1 as well as stability (Table 5). HWf and HRSf broke from their Farinograph patterns. HWf exhibited increases in C1 and ST with bran addition, while HRSf showed an increase in C1 and a decrease in ST. Overall, the results indicated that bran may have a reinforcing effect on HRW and HW doughs, whereas it is likely more destabilizing in HRS doughs. This could be the result of different protein quantity and quality among the classes. The increase in C1 with the addition of bran to the refined flours could be due to the bran requiring a longer time to hydrate [2]. Previous Mixolab studies have reported an increase in C1 compared to refined flour when coarse bran was added [2,12,13]. The addition of coarse bran to refined flour has both decreased ST [2,13] and increased it [12].

Bran particle size distribution generally did not appear to affect C1 and ST (Table 5). Alternatively, C1 has been found to decrease with a reduction in WWF particle size [2,12,13]. This has been explained as the faster absorption of water by bran at a finer particle size [2,13]. Mixolab ST has been previously reported to increase with a decrease in particle size [2,12,13]. The HRS WWFs followed this trend with HRSb53 exhibiting a significantly longer stability than the other WWFs with larger particle sizes.

C2 is a low torque point in the curve that occurs when heating is initiated (Figure 2A–C). It typically occurs around ~50 °C before starch begins to gelatinize, and it provides information on gluten strength through thermally-induced restructuring of low energy interactions (i.e., hydrogen bonds and/or hydrophobic interactions) [47]. The HWf and HRSf refined flours grouped closely in terms of C2 values (Table 5), revealing that there were few differences in terms of gluten softening on heating. The HRWf had a slightly greater C2 value indicating stronger gluten compared to HWf and HRSf. HW, HRW, and HRS WWF C2 values increased by similar amounts compared to their refined flour controls (Table 5). Therefore, the C2 values for HRW WWF were greater compared to HW and HRS WWF, which suggests that HRW WWF had increased gluten strength. Wang et al. [2] also reported an increase in WWF C2 values compared to the refined flour control. Bressiani et al. [48] hypothesized that the added fiber resulted in gluten dilution and lower gluten yield due to less aggregation of gluten proteins. The lower gluten yield could result in less overall weakening and therefore a higher C2 torque [48]. A reduction in particle size did not have an effect on C2, which is in agreement with a previous study [2].

Points C3, C4, and C5 are related to starch pasting properties (Figure 2A–C). C3 is the peak of the torque curve during heating and is related to starch gelatinization and pasting properties. Again, all three wheat classes clustered between 1.68–2.09 Nm (Table 5). HW and HRW starch appeared to be the least affected by the presence of bran. Indeed, the C3 value was almost constant across the whole HW and HRW sample set. There were only slight increases in C3 for HW and HRW starch upon the addition of bran. HRS showed a significant increase in C3 with the addition of bran (Table 5). This could be due to additional gel formation and the interaction between water and flour components [48]. In contrast, previous studies have reported WWF to have lower C3 values compared to refined flour [2,12,13]. There was no particle size effect on C3 in agreement with Wang et al. [2] and Liu et al. [13], although Xu et al. [12] reported that C3 increased with a decrease in WWF particle size.

C4 is the lowest torque point after the C3 peak, and C3–C4 is regarded as an indication of amylolytic activity and hot gel stability related to the shear thinning of the dough after starch gelatinization (Figure 2A–C). The grain samples in this study all exhibited Falling Number values above the 300 s threshold, and no exogenous amylase was added after milling. Therefore, amylase activity should not be a major factor influencing C3–C4 values. A lower C3–C4 value indicates a more stable starch gel. C3–C4 values from high to low were HRS > HRW > HW (Table 5). The wheat class differences show HW to be more resistant to shear thinning than HRW and HRS. Liu et al. [13] evaluated HW, HRW, and HRS reconstituted flours using the Mixolab and found that HW did not have significantly better hot gel stability than the other classes. This shows that results for a particular wheat class may not translate across all varieties, growing regions, and crop years. Bran particle size did not affect C3–C4 values (Table 5). Previous Mixolab studies have shown that a decrease in WWF particle size resulted in a decrease in C3–C4, indicating a more stable starch gel [2,12,13].

Retrogradation potential is indicated by the C5 value, also called setback in starch pasting terminology (Figure 2A–C). This is the maximum torque at the end of the test after a full heating and cooling cycle. HW exhibited the greatest setback value, meaning that its amylose more quickly reassociates after gelatinization (Table 5). This has implications for the texture and shelf-life of products, with greater setbacks generally leading to firmer textures and greater rates of staling (i.e., shorter shelf-life). It might be expected, then, that HW products will potentially stale faster than HRS or HRW products. Liu et al. [13] found that HW reconstituted WWF trended towards greater, although not always statistically significant, C5 values compared to HRW and HRS reconstituted WWF. This trend may extend to other HW varieties, growing regions, and crop years. WWF particle size did not affect C5 values (Table 5), although a decrease in WWF particle size has been reported to result in an increase in C5 in several studies [2,12,13].

### 3.6. Extensibility of Steamed Bread Doughs

Table 6 displays the extensibility results of full formula steamed bread dough from refined flours and reconstituted WWF. HRSf showed the greatest extensibility among the control flours. Although HWf and HRWf displayed less extensibility than HRSf, they were more similar to one other and in the same general range as HRSf. The addition of bran reduced extensibility across all wheat classes compared to the control refined flours, which aligns with previous findings [9,15,42,49,50]. In contrast, Lin et al. [16] reported that fine WWF had a greater extensibility than the refined control. Bran is known to disrupt the gluten network, which reduces dough extensibility [2,16]. The introduction of localized weak spots may also negatively affect the formation of gluten [18]. These factors result in premature rupturing of the dough during extensibility testing [2,16].

Within the WWFs, HRS had the greatest extensibility (Table 6). In the cases of HRW and HW, the bran particle size did not appear to play a large role in the extent of extensibility loss (Table 6). However, HRS showed a trend of greater extensibility loss as bran particle size increased, likely as a result of larger bran particles introducing larger localized disruptions of the gluten network. In agreement with the HRS WWF trend, extensibility has been reported to decrease as WWF particle size increased [2,16,42]. Like HRW and HW WWF results, Zhang and Moore [15] reported that extensibility did not change with particle size. The general lack of response to bran particle size in the HW and HRW samples may be a function of protein content and/or gluten relaxation kinetics in the presence of bran.

The resistance to extension is generally used as a proxy for gluten strength in flour + water doughs and an indicator of gas-holding capacity, although it is not as straightforward in full formula doughs. Resistance to extension has been reported to decrease with the addition of wheat bran compared to a refined flour dough [2,15,50]. This was not the case for all wheat classes and particle sizes as shown in Table 6. HWf stood out among the control flours for displaying the least resistance to extension. This is interesting given its apparent strength in Farinograph and Mixolab tests. HRSf was expected to exhibit the greatest resistance to extension through a combination of having the greatest protein content and strength. However, in this dough formula, it falls between HWf and HRWf. Being the weakest of the control flour samples in Farinograph testing, it was surprising to see HRWf provide the greatest resistance to extension. However, HRWf did have the greatest Mixolab stability and C2 value of the control flours, indicating gluten strength. The departure of the three wheat classes from expected trends is likely a result of ingredient interactions in the full formula dough.

Interestingly, HRW WWF samples still stand out from HRS and HW samples in terms of bran particle size trends. A reduction in bran particle size resulted in an increase in HRW resistance to extension. The HRW WWF samples aligned with previous findings that the resistance to extension increased as WWF particle size decreased [2,15,16,42]. HW and HRS WWF exhibited the opposite trend, in this case exhibiting less resistance to extension with decreasing bran particle size. In general, it may be that smaller bran particles create multiple small points of weakness in the gluten network, and these may add up to a greater decrease in overall resistance. The reduction of WWF particle size has been thought to increase the surface area for more interactions with gluten resulting in detrimental effects on the gluten network [51]. It is unclear why the classes exhibited different trends with bran particle size, although HRW samples also deviated from HRS and HW samples in Farinograph and Mixolab characteristics. Thus, it seems likely to be related to gluten protein characteristics based on overall class genetics.

The ratio of resistance to extensibility (R/E) value shows the balance of elastic to viscous properties of the dough after relaxation. The larger the R/E value, the stronger the gluten and/or less the extensibility [52]. The HRW samples showed the greatest R/E values compared to the HRS and HW samples (Table 6). The HRS samples maintained slightly more extensibility relative to a similar amount of strength compared to HRW. The HW samples showed more extensibility and less resistance to extension compared to HRW. An optimal R/E value for whole wheat steamed bread has not been identified. Steamed bread with optimal volume and even internal crumb structure needs to have gluten elasticity to prevent the rupture of gas cells and give bread with large appealing volume and smooth internal structure [2,16]. In addition to elasticity, adequate extensibility is required to allow for the expansion of gas cells during proofing and steaming [2].

### 3.7. Steamed Bread Specific Volume

Table 7 shows the specific volume data for steamed breads made from refined flours and reconstituted WWF. The order of specific volumes for steamed breads from the control flours was HWf > HRSf > HRWf (2.92, 2.61, and 2.48 cm^3^/g, respectively). These differences were not statistically significant within this data set. The addition of bran universally resulted in decreased specific volume across all classes for Chinese steamed bread, which is consistent with previous findings [2,12,49,50]. The addition of wheat bran can disrupt the gluten network through physical (shearing) and chemical mechanisms, cause fiber-gluten interactions [2,51], dilute the gluten proteins [2,53] and inhibit flour protein aggregation [2], which all weaken the dough structure. These factors disrupt the gas cell framework, which impairs gas retention and gas cell expansion, resulting in lower specific volume [2,16,53].

**Figure 3:**
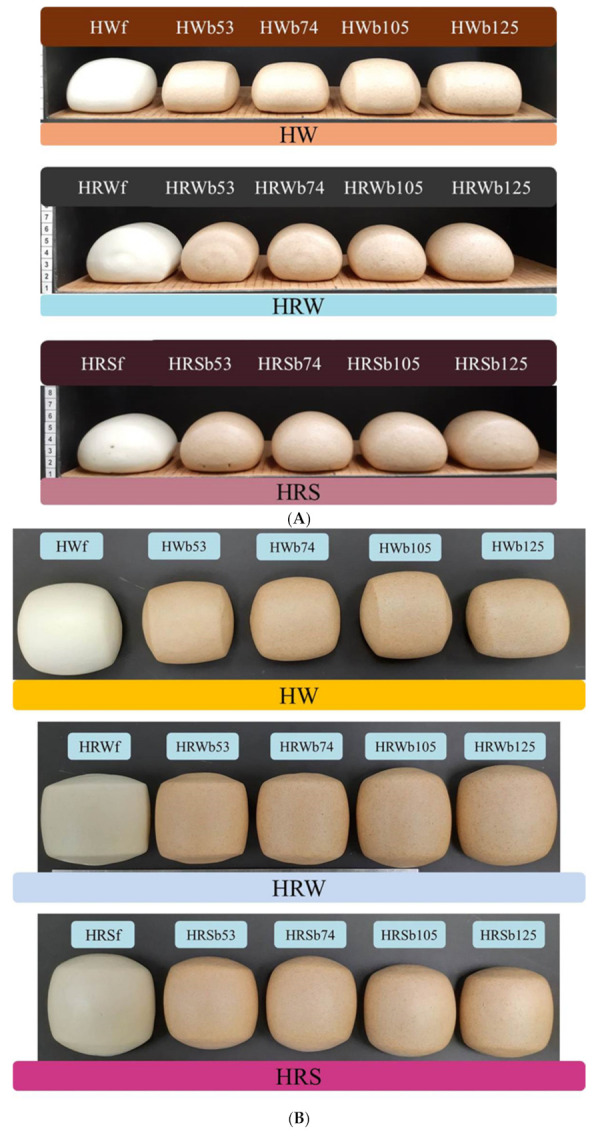

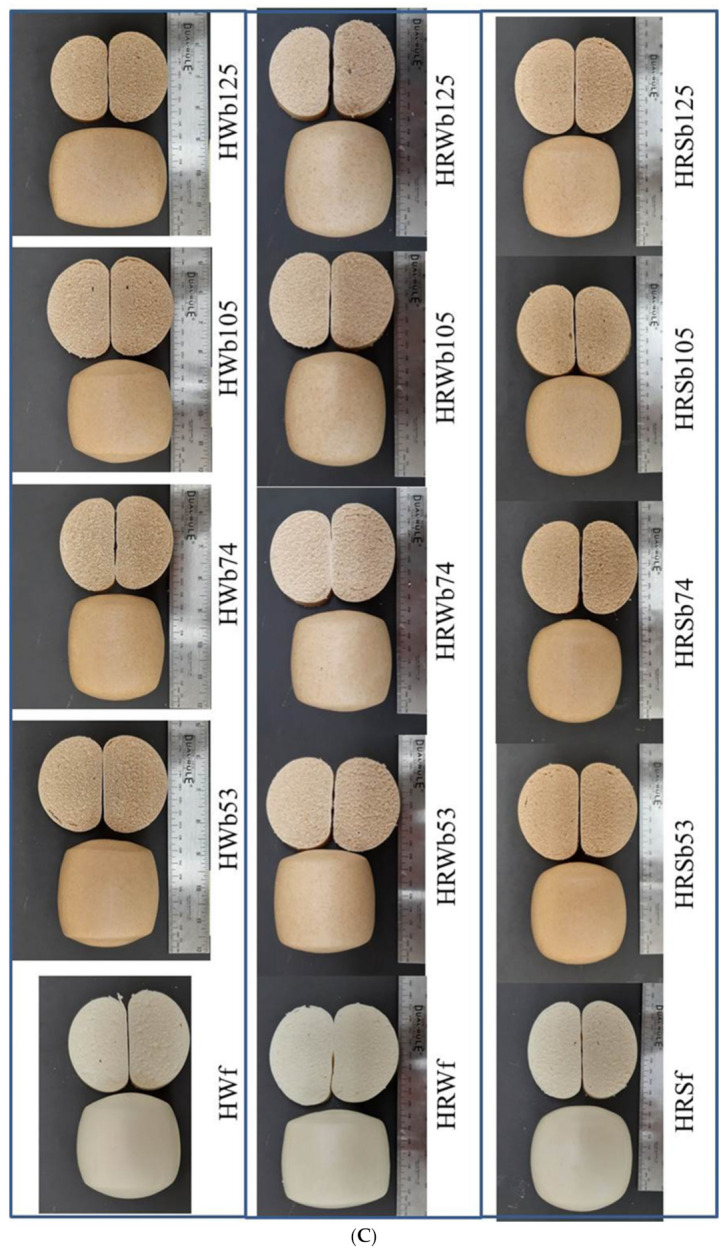
Steamed breads made from refined flours and reconstituted WWF of HW, HRW, and HRS wheat classes. (**A**) Side profiles; (**B**) top surfaces; and (**C**) crumb cross-sections. HWf, HRWf, and HRSf were 100% refined straight grade control flours. Reconstituted WWF contained 85% refined control flour and 15% bran (*w*/*w*). Reconstituted WWF: HWb53, HRWb53, and HRSb53 with an average bran particle size of 53 μm; HWb74, HRWb74, and HRSb74 with an average bran particle size of 74 μm; HWb105, HRWb105, and HRSb105 with an average bran particle size of 105 μm; HWb125, HRWb125, and HRSb125 with an average bran particle size of 125 μm.

A clear trend was also observed for bran particle size, with smaller bran particle sizes exhibiting a smaller specific volume (Table 7). This trend can also be clearly seen in Figure 3A–C, which shows the exterior and interior of the northern steamed breads from each refined flour and reconstituted WWF. The effect of WWF particle size on streamed bread specific volume is varied. WWF particle size has been found to not affect streamed bread specific volume [14]. In agreement with this study’s results, it has also been reported that decreasing wheat bran particle size decreased specific volume for both NTSB and STSB [12]. In contrast, coarse WWF produced STSB with smaller specific volume [2].

When compared with the specific volume of their respective control flours, HWb125 decreased by 16.4%, HRWb125 decreased by 6.0%, and HRSb125 decreased by 11.9%, respectively (Table 7). Steamed bread made with a bran particle size distribution mean of 125 μm was able to achieve larger specific volumes across all wheat classes when compared to the other bran particle size distributions. Therefore, after evaluating all the results, it can be understood that bran with a smaller particle size may be more likely to affect the formation of the gluten network structure during dough mixing and thereby decrease extensibility and gas retention of the dough to the extent that the specific volume of the end product is more negatively affected. Interestingly, the R/E ratios were tightly grouped (1.35–1.48) for each of the 125 µm bran samples (Table 6). It’s possible that this particle size affected the gluten network in such a way that there was more adequate gluten elasticity and extensibility to give the largest specific volume.

Another factor that could have influenced specific volume is related to the Su-SRC results. The Su-SRC values increased as bran particle size decreased, indicating increased accessibility of AX by the solvent (Table 3). This could be due to more broken cell walls in the bran fraction as the particle size was reduced. Li et al. [53] reported that a reduction in WWF particle size released ferulic acid from AX found in the cell walls, which improved the ability of arabinoxylan gels to compete with gluten for water. The migration of water from the gluten network to AX gels was reported to negatively affect gluten network formation and therefore result in poor baking performance of whole wheat bread [53]. This mechanism could be a factor influencing specific volume results at smaller bran particle size distributions. It is worth noting that reducing agents such as glutathione, which is present in greater concentrations in the bran, may also have negative effects on gluten functionality as bran particle size decreases [51].

### 3.8. Steamed Bread Color

Table 8 shows the *L*, *a*, *b*, and whiteness index (WI) for the steamed breads. In all cases, the reconstituted WWF *L* and WI were lower than those of the control flours. Smaller bran particle sizes resulted in lower *L* and WI values than those from larger bran particle sizes. This may be related to differences in specific volume as larger volumes tend to result in brighter, whiter colors. The effect of specific volume is the most likely explanation for the differences in steamed bread color as WWF with a fine particle size has been reported to have a lighter color than coarse ground WWF [14].

### 3.9. Steamed Bread Texture Profile Analysis

The textural characteristics of steamed breads as determined by TPA are shown in Table 9. All steamed breads from reconstituted WWF showed greater firmness and chewiness than their respective refined flours, which is consistent with previous reports for CSB [2,12,50]. The HRW samples displayed a trend towards increasing firmness and chewiness as bran particle size decreased, which loosely aligned with specific volume (Table 7) and resistance to extension trends (Table 6). However, neither the HW nor the HRS groups demonstrated such a consistent trend in terms of bran particle size and texture. There have been inconsistent findings related to the relationship between bran particle size and texture. Xu et al. [12] reported that decreasing WWF particle size resulted in firmer crumbs and increased chewiness for both STSB and NTSB. In contrast, Wang et al. [2] found that coarse WWF produced Chinese STSB with a firmer texture and increased chewiness. While HWb125, HRWb125, and HRSb125 all showed larger specific volumes among the reconstituted WWF steamed breads (Table 7), there was no significant correlation to the texture values (Table 9).

The greatest firmness was observed in HW and HRW samples. The protein content of HRSf was 15.66%. Adding bran or other materials in refined flour affects the formation of the gluten network during dough mixing. For refined flour with lower protein content, bran can have a more negative effect on the end product. It can therefore be speculated that HRS, with its naturally greater protein content and strong, extensible gluten characteristics, is more capable of overcoming the deleterious impact of bran in whole wheat steamed bread than either HW or HRW.

## 4. Conclusions

Other research has shown that smaller bran particle sizes can adversely affect bread quality. This study presents similar findings, with larger bran particle size distributions (105 μm and 125 μm) being more suitable for producing whole-wheat steamed bread with a larger specific volume, brighter color, and softer texture. HRS appeared to be the most suitable wheat class for whole wheat steamed bread across the majority of the instrumentally measured product characteristics, although this would need to be confirmed with a sensory study.

From a flour quality and dough rheology standpoint, none of the tests performed in this study offered sufficient predictive potential related to final product quality. The SRC GPI offered some promise in predicting steamed bread volume, with values approaching ~0.80 leading to better specific volumes. The most promising rheological method appeared to be dough extensibility and resistance to extension, with R/E values approaching ~1.50 being more conducive to optimal specific volume and texture (hardness) of whole wheat steamed bread for a given wheat class. However, this needs to be investigated with a larger sample set encompassing a wider range of flour quality characteristics.

Future work should focus on investigating large bran particle sizes across a wider range of samples to identify optimal wheat quality, flour quality, and dough rheology parameters for CSB, as well as new methodology that is optimized for WWF.

## Figures and Tables

**Table 1:** Composition and damaged starch content of refined flour and reconstituted WWF.

Sample	Moisture	Protein	Ash	Damaged Starch (SD-Matic)
	%	Dry Basis (db)		%
Bran(125μm)	6.69 ± 0.20	15.79 ± 0.02	6.00 ± 0.01	- -
HWf	13.75 ± 0.01 ^a^	13.77 ± 0.04 ^c^	0.55 ± 0.01 ^c^	3.23 ± 0.12 ^e^
HWb53	12.49 ± 0.01 ^ef^	13.65 ± 0.03 ^bc^	1.46 ± 0.05 ^a^	3.70 ± 0.12 ^d^
HWb74	12.56 ± 0.02 ^e^	13.85 ± 0.05 ^bc^	1.40 ± 0.01 ^ab^	3.59 ± 0.04 ^d^
HWb105	12.71 ± 0.04 ^d^	13.95 ± 0.04 ^b^	1.43 ± 0.02 ^ab^	3.39 ± 0.11 ^de^
HWb125	12.74 ± 0.04 ^d^	13.81 ± 0.09 ^bc^	1.43 ± 0.01 ^ab^	3.49 ± 0.17 ^de^
HRWf	13.00 ± 0.04 ^c^	12.36 ± 0.32 ^e^	0.61 ± 0.01 ^c^	5.92 ± 0.07 ^a^
HRWb53	12.25 ± 0.11 ^h^	12.77 ± 0.01 ^d^	1.36 ± 0.04 ^b^	5.45 ± 0.17 ^b^
HRWb74	12.37 ± 0.04 ^g^	12.63 ± 0.15 ^d^	1.36 ± 0.05 ^b^	5.74 ± 0.14 ^b^
HRWb105	12.50 ± 0.05 ^ef^	12.80 ± 0.04 ^d^	1.36 ± 0.03 ^b^	5.92 ± 0.26 ^ab^
HRWb125	12.42 ± 0.02 ^fg^	12.66 ± 0.10 ^d^	1.41 ± 0.03 ^ab^	5.65 ± 0.25 ^b^
HRSf	13.17 ± 0.08 ^b^	15.66 ± 0.02 ^a^	0.59 ± 0.09 ^c^	4.35 ± 0.12 ^c^
HRSb53	12.44 ± 0.01 ^fg^	15.67 ± 0.06 ^a^	1.36 ± 0.00 ^b^	4.23 ± 0.11 ^c^
HRSb74	12.52 ± 0.04 ^ef^	15.81 ± 0.22 ^a^	1.40 ± 0.02 ^ab^	4.23 ± 0.13 ^c^
HRSb105	12.59 ± 0.01 ^e^	15.74 ± 0.16 ^a^	1.41 ± 0.03 ^ab^	4.27 ± 0.05 ^c^
HRSb125	12.57 ± 0.02 ^e^	15.77 ± 0.04 ^a^	1.39 ± 0.00 ^ab^	4.04 ± 0.10 ^c^

*n* = 3. Values followed by the different letters in the same column are significantly different (*p* < 0.05). Whole wheat flour (WWF), hard white (HW), hard red winter (HRW), and hard red spring (HRS) wheat classes. HWf, HRWf, and HRSf were 100% refined straight grade control flours. Reconstituted WWF contained 85% refined control flour and 15% bran (*w*/*w*). Reconstituted WWF: HWb53, HRWb53, and HRSb53 with an average bran particle size of 53 μm; HWb74, HRWb74, and HRSb74 with an average bran particle size of 74 μm; HWb105, HRWb105, and HRSb105 with an average bran particle size of 105 μm; HWb125, HRWb125, and HRSb125 with an average bran particle size of 125 μm.

**Table 2:** Damaged starch content of refined flour and reconstituted WWF as measured by the SD-matic and Megazyme methods.

	SD-Matic	Megazyme
	%	
HWf	3.23 ± 0.12 ^e^	4.04 ± 1.06 ^f^
HWb53	3.70 ± 0.12 ^d^	5.17 ± 0.10 ^de^
HWb74	3.59 ± 0.04 ^d^	5.08 ± 0.04 ^e^
HWb105	3.39 ± 0.11 ^de^	4.99 ± 0.28 ^e^
HWb125	3.49 ± 0.17 ^de^	4.98 ± 0.16 ^e^
HRWf	5.92 ± 0.07 ^a^	7.20 ± 0.47 ^a^
HRWb53	5.45 ± 0.17 ^b^	6.55 ± 0.20 ^b^
HRWb74	5.74 ± 0.14 ^b^	6.62 ± 0.08 ^b^
HRWb105	5.92 ± 0.26 ^ab^	6.73 ± 0.15 ^b^
HRWb125	5.65 ± 0.25 ^b^	6.58 ± 0.15 ^b^
HRSf	4.35 ± 0.12 ^c^	5.71 ± 0.09 ^c^
HRSb53	4.23 ± 0.11 ^c^	5.62 ± 0.09 ^cd^
HRSb74	4.23 ± 0.13 ^c^	5.61 ± 0.10 ^cd^
HRSb105	4.27 ± 0.05 ^c^	5.65 ± 0.04 ^c^
HRSb125	4.04 ± 0.10 ^c^	5.57 ± 0.22 ^cd^

*n* = 3. Values followed by the different letters in the same column are significantly different (*p* < 0.05). Whole wheat flour (WWF), hard white (HW), hard red winter (HRW), and hard red spring (HRS) wheat classes. HWf, HRWf, and HRSf were 100% refined straight grade control flours. Reconstituted WWF contained 85% refined control flour and 15% bran (*w*/*w*). Reconstituted WWF: HWb53, HRWb53, and HRSb53 with an average bran particle size of 53 μm; HWb74, HRWb74, and HRSb74 with an average bran particle size of 74 μm; HWb105, HRWb105, and HRSb105 with an average bran particle size of 105 μm; HWb125, HRWb125, and HRSb125 with an average bran particle size of 125 μm.

**Table 3:** SRC profiles of refined flour and reconstituted WWF.

	Water	Lactic Acid	Sucrose	Sodium Carbonate	GPI *
	%, As-Is				
HWf	58.6 ± 0.7 ^f^	155.3 ± 8.9 ^c^	102.2 ± 2.4 ^g^	71.7 ± 0.7 ^f^	0.89 ± 0.05 ^c^
HWb53	70.1 ± 0.9 ^b–d^	116.0 ± 2.0 ^h^	111.6 ± 1.7 ^e^	80.6 ± 1.0 ^de^	0.60 ± 0.01 ^i^
HWb74	69.5 ± 2.8 ^b–d^	116.0 ± 2.1 ^h^	112.2 ± 2.1 ^e^	79.4 ± 0.6 ^e^	0.61 ± 0.01 ^hi^
HWb105	68.6 ± 0.4 ^d^	118.2 ± 1.0 ^h^	111.4 ± 1.6 ^e^	80.2 ± 1.3 ^e^	0.62 ± 0.01 ^hi^
HWb125	68.7 ± 1.4 ^d^	118.2 ± 2.3 ^h^	107.5 ± 1.5 ^f^	79.8 ± 0.9 ^e^	0.63 ± 0.01 ^hg^
HRWf	62.0 ± 0.7 ^e^	199.9 ± 1.7 ^b^	116.2 ± 1.5 ^d^	84.6 ± 0.6 ^c^	1.00 ± 0.01 ^b^
HRWb53	72.7 ± 1.8 ^a^	138.3 ± 1.5 ^e–g^	120.2 ± 1.9 ^ab^	88.7 ± 1.0 ^b^	0.66 ± 0.01 ^ef^
HRWb74	71.4 ± 1.3 ^a–c^	134.6 ± 2.3 ^fg^	116.6 ± 0.8 ^d^	90.5 ± 1.0 ^a^	0.65 ± 0.01 ^fg^
HRWb105	71.3 ± 1.3 ^a–c^	136.3 ± 1.9 ^e–g^	117.9 ± 1.6 ^b–d^	88.7 ± 0.5 ^b^	0.66 ± 0.01 ^ef^
HRWb125	69.4 ± 0.8 ^cd^	133.8 ± 3.4 ^g^	117.0 ± 2.0 ^d^	87.9 ± 0.9 ^b^	0.65 ± 0.01 ^fg^
HRSf	61.1 ± 0.9 ^e^	208.5 ± 3.2 ^a^	110.5 ± 1.6 ^e^	81.9 ± 1.3 ^d^	1.09 ± 0.01 ^a^
HRSb53	71.5 ± 1.6 ^ab^	142.4 ± 3.5 ^d^	121.5 ± 2.1 ^a^	87.4 ± 1.7 ^b^	0.68 ± 0.02 ^de^
HRSb74	70.4 ± 1.0 ^ab^	139.7 ± 3.6 ^d–f^	119.9 ± 1.5 ^a–c^	87.5 ± 0.4 ^b^	0.68 ± 0.02 ^d–f^
HRSb105	70.4 ± 0.8 ^b–d^	140.5 ± 1.6 ^de^	117.5 ± 1.3 ^cd^	88.7 ± 1.2 ^b^	0.68 ± 0.01 ^de^
HRSb125	69.3 ± 0.9 ^cd^	141.5 ± 2.6 ^de^	116.7 ± 1.5 ^d^	87.8 ± 0.7 ^b^	0.69 ± 0.02 ^d^

*n* = 3. Values followed by the different letters in the same column are significantly different (*p* < 0.05). * Gluten performance index (GPI) = Lactic acid SRC/(Sodium carbonate SRC + Sucrose SRC). Whole wheat flour (WWF), hard white (HW), hard red winter (HRW), and hard red spring (HRS) wheat classes. HWf, HRWf, and HRSf were 100% refined straight grade control flours. Reconstituted WWF contained 85% refined control flour and 15% bran (*w*/*w*). Reconstituted WWF: HWb53, HRWb53, and HRSb53 with an average bran particle size of 53 μm; HWb74, HRWb74, and HRSb74 with an average bran particle size of 74 μm; HWb105, HRWb105, and HRSb105 with an average bran particle size of 105 μm; HWb125, HRWb125, and HRSb125 with an average bran particle size of 125 μm.

**Table 4:** Farinograph mixing properties for refined flour and reconstituted WWF.

	WA	DT	ST
	%, As-Is	Min	Min
HWf	56.2 ± 0.0 ^k^	5.9 ± 0.1 ^b^	9.7 ± 1.0 ^c–e^
HWb53	64.3 ± 0.1 ^d^	4.5 ± 0.3 ^de^	5.7 ± 0.1 ^e^
HWb74	63.5 ± 0.0 ^g^	4.4 ± 0.1 ^de^	8.4 ± 1.2 ^b–e^
HWb105	63.2 ± 0.0 ^h^	4.7 ± 0.0 ^de^	8.3 ± 1.9 ^b–e^
HWb125	63.2 ± 0.0 ^h^	4.2 ± 0.5 ^e^	6.0 ± 1.3 ^de^
HRWf	58.1 ± 0.2 ^g^	2.2 ± 0.2 ^f^	8.1 ± 0.5 ^c–e^
HRWb53	64.6 ± 0.1 ^c^	4.3 ± 0.6 ^e^	9.5 ± 1.3 ^a–c^
HRWb74	64.2 ± 0.0 ^de^	5.2 ± 0.0 ^b–d^	11.5 ± 2.0 ^ab^
HRWb105	63.8 ± 0.0 ^f^	6.0 ± 0.3 ^b^	12.2 ± 0.4 ^a^
HRWb125	64.0 ± 0.0 ^ef^	4.7 ± 0.2 ^de^	11.0 ± 0.6 ^a–c^
HRSf	61.0 ± 0.0 ^i^	7.4 ± 0.2 ^a^	11.0 ± 1.5 ^a–c^
HRSb53	67.4 ± 0.0 ^a^	5.6 ± 0.6 ^bc^	8.9 ± 1.9 ^a–e^
HRSb74	67.5 ± 0.0 ^a^	5.0 ± 0.8 ^c–e^	9.2 ± 0.6 ^b–e^
HRSb105	67.1 ± 0.4 ^b^	5.3 ± 0.1 ^b–d^	8.1 ± 2.8 ^b–e^
HRSb125	67.0 ± 0.0 ^b^	5.2 ± 0.4 ^b–d^	9.1 ± 1.1 ^a–e^

*n* = 3. Values followed by the different letters in the same column are significantly different (*p* < 0.05). Water absorption (WA), dough development time (DT), and mixing stability (ST). Whole wheat flour (WWF), hard white (HW), hard red winter (HRW), and hard red spring (HRS) wheat classes. HWf, HRWf, and HRSf were 100% refined straight grade control flours. Reconstituted WWF contained 85% refined control flour and 15% bran (*w*/*w*). Reconstituted WWF: HWb53, HRWb53, and HRSb53 with an average bran particle size of 53 μm; HWb74, HRWb74, and HRSb74 with an average bran particle size of 74 μm; HWb105, HRWb105, and HRSb105 with an average bran particle size of 105 μm; HWb125, HRWb125, and HRSb125 with an average bran particle size of 125 μm.

**Table 5:** Mixolab characteristics of refined flours and reconstituted WWF.

	C1	Stability	C2	C3	C4	C3–C4	C5
	Min		Nm				
HWf	5.02 ± 0.05 ^f^	9.05 ± 0.07 ^de^	0.46 ± 0.01 ^g^	1.98 ± 0.01 ^c^	1.94 ± 0.01 ^a^	0.04 ± 0.01 ^e^	4.05 ± 0.07 ^a^
HWb53	7.62 ± 0.37 ^bc^	9.65 ± 0.07 ^bc^	0.52 ± 0.00 ^ef^	2.03 ± 0.00 ^b^	1.93 ± 0.00 ^a^	0.10 ± 0.01 ^de^	4.13 ± 0.04 ^a^
HWb74	7.60 ± 0.21 ^bc^	9.79 ± 0.16 ^bc^	0.51 ± 0.00 ^f^	2.02 ± 0.00 ^bc^	1.92 ± 0.01 ^ab^	0.10 ± 0.01 ^de^	4.10 ± 0.07 ^a^
HWb105	6.90 ± 0.03 ^e^	9.55 ± 0.21 ^bc^	0.52 ± 0.01 ^ef^	2.03 ± 0.02 ^b^	1.91 ± 0.02 ^ab^	0.12 ± 0.00 ^d^	4.12 ± 0.05 ^a^
HWb125	7.29 ± 0.13 ^cd^	9.55 ± 0.07 ^bc^	0.53 ± 0.01 ^de^	2.02 ± 0.01 ^bc^	1.88 ± 0.01 ^b^	0.14 ± 0.01 ^d^	4.05 ± 0.05 ^a^
HRWf	1.60 ± 0.04 ^g^	9.95 ± 0.07 ^b^	0.54 ± 0.00 ^d^	2.05 ± 0.07 ^ab^	1.66 ± 0.03 ^e^	0.40 ± 0.01 ^a^	3.20 ± 0.06 ^d^
HRWb53	8.09 ± 0.01 ^a^	10.84 ± 0.08 ^a^	0.61 ± 0.01 ^a^	2.09 ± 0.01 ^a^	1.76 ± 0.01 ^c^	0.34 ± 0.02 ^abc^	3.61 ± 0.04 ^b^
HRWb74	7.95 ± 0.04 ^ab^	10.99 ± 0.01 ^a^	0.58 ± 0.00 ^c^	2.09 ± 0.00 ^a^	1.71 ± 0.01 ^d^	0.38 ± 0.01 ^a^	3.48 ± 0.04 ^c^
HRWb105	7.80 ± 0.31 ^ab^	10.89 ± 0.01 ^a^	0.59 ± 0.01 ^b^	2.09 ± 0.01 ^a^	1.73 ± 0.00 ^cd^	0.36 ± 0.02 ^ab^	3.59 ± 0.02 ^b^
HRWb125	8.05 ± 0.04 ^a^	10.65 ± 0.21 ^a^	0.59 ± 0.00 ^bc^	2.07 ± 0.00 ^ab^	1.71 ± 0.02 ^d^	0.35 ± 0.02 ^ab^	3.55 ± 0.04 ^bc^
HRSf	6.94 ± 0.08 ^de^	9.60 ± 0.28 ^bc^	0.44 ± 0.00 ^h^	1.68 ± 0.00 ^e^	1.41 ± 0.02 ^g^	0.27 ± 0.02 ^c^	2.70 ± 0.04 ^e^
HRSb53	8.09 ± 0.05 ^a^	9.35 ± 0.35 ^cd^	0.51 ± 0.01 ^f^	1.84 ± 0.02 ^d^	1.55 ± 0.02 ^f^	0.29 ± 0.00 ^bc^	3.14 ± 0.02 ^d^
HRSb74	7.68 ± 0.32 ^abc^	8.85 ± 0.04 ^e^	0.51 ± 0.00 ^f^	1.83 ± 0.02 ^d^	1.56 ± 0.03 ^f^	0.27 ± 0.05 ^c^	3.13 ± 0.06 ^d^
HRSb105	8.04 ± 0.06 ^a^	8.85 ± 0.49 ^e^	0.54 ± 0.00 ^d^	1.85 ± 0.01 ^d^	1.55 ± 0.00 ^f^	0.30 ± 0.01 ^bc^	3.17 ± 0.01 ^d^
HRSb125	7.89 ± 0.16 ^ab^	8.82 ± 0.26 ^e^	0.53 ± 0.01 ^de^	1.82 ± 0.00 ^d^	1.55 ± 0.00 ^f^	0.28 ± 0.00 ^c^	3.16 ± 0.04 ^d^

*n* = 2. Values followed by the different letters in the same column are significantly different (*p* < 0.05). C1 = Time to maximum mixing torque; C2 = Minimum torque during initial heating phase, related to protein weakening due to heating; C3 = Maximum torque during heating, related to starch pasting properties; C4 = Minimum torque during heating, related to amylase activity and starch stability; C5 = Maximum torque during cooling, related to starch retrogradation. Whole wheat flour (WWF), hard white (HW), hard red winter (HRW), and hard red spring (HRS) wheat classes. HWf, HRWf, and HRSf were 100% refined straight grade control flours. Reconstituted WWF contained 85% refined control flour and 15% bran (*w*/*w*). Reconstituted WWF: HWb53, HRWb53, and HRSb53 with an average bran particle size of 53 μm; HWb74, HRWb74, and HRSb74 with an average bran particle size of 74 μm; HWb105, HRWb105, and HRSb105 with an average bran particle size of 105 μm; HWb125, HRWb125, and HRSb125 with an average bran particle size of 125 μm.

**Table 6:** Extensibility of steamed bread dough made from refined flour and reconstituted WWF.

	Resistance to Extension	Extensibility	R/E
	g	mm	
HWf	19.61 ± 2.11 ^g^	41.83 ± 10.03 ^b^	0.51 ± 0.24 ^g^
HWb53	20.76 ± 1.81 ^g^	19.76 ± 2.64 ^gh^	1.06 ± 0.13 ^de^
HWb74	20.54 ± 1.46 ^g^	19.88 ± 3.64 ^gh^	1.07 ± 0.22 ^de^
HWb105	23.85 ± 2.64 ^f^	21.83 ± 10.41 ^gf^	1.18 ± 0.26 ^d^
HWb125	25.70 ± 1.56 ^de^	19.45 ± 3.12 ^gh^	1.35 ± 0.18 ^c^
HRWf	30.66 ± 4.00 ^b^	38.17 ± 6.95 ^c^	0.85 ± 0.30 ^g^
HRWb53	33.22 ± 1.69 ^a^	17.59 ± 1.40 ^h^	1.90 ± 0.19 ^a^
HRWb74	26.17 ± 3.76 ^de^	17.40 ± 2.25 ^h^	1.54 ± 0.37 ^b^
HRWb105	25.83 ± 2.21 ^de^	17.65 ± 3.43 ^h^	1.53 ± 0.43 ^b^
HRWb125	25.85 ± 2.16 ^de^	17.95 ± 3.64 ^gh^	1.48 ± 0.23 ^bc^
HRSf	28.37 ± 3.76 ^c^	47.22 ± 5.81 ^a^	0.61 ± 0.14 ^g^
HRSb53	25.27 ± 1.38 ^d–f^	29.74 ± 4.15 ^d^	0.86 ± 0.11 ^f^
HRSb74	25.14 ± 2.18 ^ef^	28.07 ± 4.71 ^de^	0.92 ± 0.18 ^ef^
HRSb105	27.06 ± 0.93 ^cd^	26.32 ± 2.74 ^de^	1.04 ± 0.11 ^de^
HRSb125	34.74 ± 3.19 ^a^	24.53 ± 2.07 ^ef^	1.43 ± 0.18 ^bc^

*n* = 3. Values followed by the different letters in the same column are significantly different (*p* < 0.05). Whole wheat flour (WWF), hard white (HW), hard red winter (HRW), and hard red spring (HRS) wheat classes. HWf, HRWf, and HRSf were 100% refined straight grade control flours. Reconstituted WWF contained 85% refined control flour and 15% bran (*w*/*w*). Reconstituted WWF: HWb53, HRWb53, and HRSb53 with an average bran particle size of 53 μm; HWb74, HRWb74, and HRSb74 with an average bran particle size of 74 μm; HWb105, HRWb105, and HRSb105 with an average bran particle size of 105 μm; HWb125, HRWb125, and HRSb125 with an average bran particle size of 125 μm.

**Table 7:** Specific volume of steamed breads made from refined flours and reconstituted WWF.

	Specific Volume	Relative Change in Specific Volume
	cm^3^/g	%
HWf	2.92 ± 0.04 ^a^	–
HWb53	2.28 ± 0.07 ^c^	−21.9
HWb74	2.16 ± 0.09 ^d^	−26.0
HWb105	2.38 ± 0.06 ^b^	−18.5
HWb125	2.44 ± 0.02 ^b^	−16.4
HRWf	2.48 ± 0.04 ^a^	–
HRWb53	2.22 ± 0.05 ^c^	−10.5
HRWb74	2.31 ± 0.06 ^b^	−6.9
HRWb105	2.27 ± 0.04 ^b^	−8.5
HRWb125	2.33 ± 0.08 ^b^	−6.0
HRSf	2.61 ± 0.04 ^a^	–
HRSb53	2.13 ± 0.06 ^d^	−18.4
HRSb74	2.14 ± 0.07 ^d^	−18.0
HRSb105	2.21 ± 0.05 ^c^	−15.3
HRSb125	2.30 ± 0.08 ^b^	−11.9

*n* = 3. Values followed by the same letter in the same column and wheat class are not significantly different (*p* < 0.05). Whole wheat flour (WWF), hard white (HW), hard red winter (HRW), and hard red spring (HRS) wheat classes. HWf, HRWf, and HRSf were 100% refined straight grade control flours. Reconstituted WWF contained 85% refined control flour and 15% bran (*w*/*w*). Reconstituted WWF: HWb53, HRWb53, and HRSb53 with an average bran particle size of 53 μm; HWb74, HRWb74, and HRSb74 with an average bran particle size of 74 μm; HWb105, HRWb105, and HRSb105 with an average bran particle size of 105 μm; HWb125, HRWb125, and HRSb125 with an average bran particle size of 125 μm.

**Table 8:** Color of steamed bread made from refined flour and reconstituted WWF.

	*L*	*a*	*b*	WI *
HWf	91.55 ± 0.65 ^a^	−2.03 ± 0.05 ^g^	17.55 ± 0.10 ^i^	80.41 ± 0.32 ^a^
HWb53	70.11 ± 0.64 ^d^	6.45 ± 0.10 ^cd^	24.13 ± 0.21 ^e^	61.05 ± 0.57 ^e^
HWb74	70.97 ± 0.86 ^cd^	6.75 ± 0.24 ^b^	24.58 ± 0.47 ^cd^	61.36 ± 0.97 ^de^
HWb105	71.75 ± 0.24 ^bc^	6.05 ± 0.08 ^e^	23.37 ± 0.12 ^f^	62.84 ± 0.25 ^c^
HWb125	68.65 ± 1.93 ^e^	6.53 ± 0.10 ^c^	23.50 ± 0.31 ^f^	60.26 ± 1.38 ^ef^
HRWf	91.12 ± 0.47 ^a^	−2.19 ± 0.03 ^g^	19.00 ± 0.50 ^g^	78.91 ± 0.60 ^b^
HRWb53	69.90 ± 0.71 ^d^	7.13 ± 0.06 ^a^	25.61 ± 0.23 ^a^	59.84 ± 0.41 ^f^
HRWb74	69.97 ± 0.79 ^d^	6.84 ± 0.15 ^b^	25.00 ± 0.12 ^b^	60.33 ± 0.68 ^ef^
HRWb105	72.06 ± 0.87 ^bc^	6.38 ± 0.11 ^cd^	24.37 ± 0.19 ^de^	62.38 ± 0.67 ^cd^
HRWb125	71.86 ± 0.59 ^bc^	6.44 ± 0.12 ^cd^	24.23 ± 0.22 ^de^	62.31 ± 0.54 ^cd^
HRSf	90.48 ± 0.29 ^a^	−1.76 ± 0.04 ^f^	18.59 ± 0.33 ^h^	79.04 ± 0.39 ^b^
HRSb53	70.77 ± 0.79 ^cd^	6.83 ± 0.16 ^b^	24.90 ± 0.23 ^bc^	61.00 ± 0.72 ^e^
HRSb74	70.27 ± 0.59 ^d^	6.74 ± 0.12 ^b^	24.61 ± 0.16 ^b–d^	60.82 ± 0.53 ^ef^
HRSb105	72.54 ± 0.54 ^b^	6.32 ± 0.13 ^d^	24.15 ± 0.22 ^e^	62.89 ± 0.53 ^c^
HRSb125	71.75 ± 1.20 ^bc^	6.45 ± 0.15 ^cd^	24.15 ± 0.16 ^e^	62.27 ± 0.97 ^cd^

*n* = 5. Values followed by the different letters in the same column are significantly different (*p* < 0.05). * Whiteness index (WI) = 100 − ((100 − *L*)^2^ + *a*^2^ + *b*^2^)^0.5^ Values followed by the same letter in the same row are not significantly different (*p* < 0.05). Whole wheat flour (WWF), hard white (HW), hard red winter (HRW), and hard red spring (HRS) wheat classes. HWf, HRWf, and HRSf were 100% refined straight grade control flours. Reconstituted WWF contained 85% refined control flour and 15% bran (*w*/*w*). Reconstituted WWF: HWb53, HRWb53, and HRSb53 with an average bran particle size of 53 μm; HWb74, HRWb74, and HRSb74 with an average bran particle size of 74 μm; HWb105, HRWb105, and HRSb105 with an average bran particle size of 105 μm; HWb125, HRWb125, and HRSb125 with an average bran particle size of 125 μm.

**Table 9:** Texture profile analysis of steamed breads made from refined flours and reconstituted WWF.

	Firmness (N)	Springiness	Cohesiveness	Chewiness (N)	Resilience
HWf	8.1 ± 0.3 ^d^	0.8 ± 0.03 ^a^	0.7 ± 0.01 ^a^	4.5 ± 0.2 ^d^	0.3 ± 0.01 ^a^
HWb53	16.6 ± 0.8 ^b^	0.8 ± 0.02 ^a^	0.6 ± 0.01 ^a^	8.3 ± 0.4 ^b^	0.2 ± 0.01 ^a^
HWb74	18.1 ± 0.8 ^a^	0.8 ± 0.01 ^a^	0.6 ± 0.03 ^a^	9.1 ± 0.2 ^a^	0.2 ± 0.01 ^a^
HWb105	14.3 ± 0.7 ^c^	0.8 ± 0.02 ^a^	0.6 ± 0.01 ^a^	7.3 ± 0.3 ^c^	0.2 ± 0.01 ^a^
HWb125	16.6 ± 0.8 ^b^	0.8 ± 0.01 ^a^	0.6 ± 0.01 ^a^	8.3 ± 0.3 ^b^	0.2 ± 0.01 ^a^
HRWf	11.2 ± 0.5 ^d^	0.8 ± 0.03 ^a^	0.7 ± 0.02 ^a^	5.9 ± 0.3 ^c^	0.3 ± 0.01 ^a^
HRWb53	18.3 ± 0.8 ^a^	0.8 ± 0.02 ^a^	0.6 ± 0.01 ^a^	7.7 ± 0.4 ^a^	0.2 ± 0.01 ^a^
HRWb74	16.0 ± 0.6 ^b^	0.8 ± 0.02 ^a^	0.6 ± 0.01 ^a^	7.4 ± 0.2 ^ab^	0.2 ± 0.01 ^a^
HRWb105	15.6 ± 0.8 ^b^	0.8 ± 0.03 ^a^	0.6 ± 0.01 ^a^	7.2 ± 0.3 ^b^	0.2 ± 0.01 ^a^
HRWb125	14.6 ± 0.6 ^c^	0.8 ± 0.02 ^a^	0.6 ± 0.01 ^a^	7.2 ± 0.4 ^b^	0.2 ± 0.01 ^a^
HRSf	7.8 ± 0.4 ^d^	0.9 ± 0.02 ^a^	0.7 ± 0.01 ^a^	4.7 ± 0.2 ^c^	0.3 ± 0.01 ^a^
HRSb53	14.6 ± 0.7 ^ab^	0.8 ± 0.02 ^a^	0.6 ± 0.01 ^a^	7.4 ± 0.3 ^b^	0.2 ± 0.02 ^a^
HRSb74	12.8 ± 0.4 ^c^	0.8 ± 0.04 ^a^	0.6 ± 0.01 ^a^	6.9 ± 0.3 ^b^	0.2 ± 0.01 ^a^
HRSb105	15.1 ± 0.7 ^a^	0.8 ± 0.01 ^a^	0.6 ± 0.01 ^a^	7.8 ± 0.2 ^a^	0.2 ± 0.01 ^a^
HRSb125	13.8 ± 0.6 ^b^	0.8 ± 0.01 ^a^	0.6 ± 0.01 ^a^	7.1 ± 0.3 ^b^	0.2 ± 0.01 ^a^

*n* = 5. Values followed by the same letter in the same column and wheat class are not significantly different (*p* < 0.05). Firmness = Peak force of compression cycle 1 in N; Springiness = Distance of compression cycle 1/Distance of compression cycle 2; Cohesiveness = Area of compression cycle 1/Area of compression cycle 2; Chewiness = Firmness × Springiness × Cohesiveness; Resilience = Upstroke area of compression cycle 1/Downstroke area of compression cycle 2. Whole wheat flour (WWF), hard white (HW), hard red winter (HRW), and hard red spring (HRS) wheat classes. HWf, HRWf, and HRSf were 100% refined straight grade control flours. Reconstituted WWF contained 85% refined control flour and 15% bran (*w*/*w*). Reconstituted WWF: HWb53, HRWb53, and HRSb53 with an average bran particle size of 53 μm; HWb74, HRWb74, and HRSb74 with an average bran particle size of 74 μm; HWb105, HRWb105, and HRSb105 with an average bran particle size of 105 μm; HWb125, HRWb125, and HRSb125 with an average bran particle size of 125 μm.

## Data Availability

Data are not available in public datasets, please contact the authors.
